# The Lymphatic System in Obesity, Insulin Resistance, and Cardiovascular Diseases

**DOI:** 10.3389/fphys.2019.01402

**Published:** 2019-11-14

**Authors:** Xinguo Jiang, Wen Tian, Mark R. Nicolls, Stanley G. Rockson

**Affiliations:** ^1^VA Palo Alto Health Care System, Palo Alto, CA, United States; ^2^Department of Medicine, Stanford University School of Medicine, Stanford, CA, United States

**Keywords:** lymphatic, LEC, insulin resistance, type 2 diabetes, atherosclerosis, myocardial infarction

## Abstract

Obesity, insulin resistance, dyslipidemia, and hypertension are fundamental clinical manifestations of the metabolic syndrome. Studies over the last few decades have implicated chronic inflammation and microvascular remodeling in the development of obesity and insulin resistance. Newer observations, however, suggest that dysregulation of the lymphatic system underlies the development of the metabolic syndrome. This review summarizes recent advances in the field, discussing how lymphatic abnormality promotes obesity and insulin resistance, and, conversely, how the metabolic syndrome impairs lymphatic function. We also discuss lymphatic biology in metabolically dysregulated diseases, including type 2 diabetes, atherosclerosis, and myocardial infarction.

## Introduction

Obesity, characterized by increased storage of fatty acids in expanded adipose tissues, is becoming a major health problem in modern society, as humans increasingly embrace a relatively sedentary lifestyle. The chronic obese status predisposes individuals to the development of the metabolic syndrome and increases the incidence of type 2 diabetes (T2D) and cardiovascular diseases (Kahn and Flier, 2000; O’Neill and O’Driscoll, 2015; Catrysse and van Loo, 2017). Dysregulated lipid metabolism and low-grade chronic inflammation are among the notable pathologies in obese adipose tissue (Ruotolo and Howard, 2002; Konner and Bruning, 2011). More recently, there is increasing evidence that dysfunction of the lymphatic vasculature is involved in the pathogenesis of obesity and obesity-associated dyslipidemia and low-grade chronic inflammation (Harvey et al., 2005; Aspelund et al., 2016), presumably because the lymphatic system is important for immune homeostasis and lipid transport (Jiang et al., 2018). This review provides an overview of the interplay between the function of lymphatic system and presence of obesity and insulin resistance. We also discuss how the lymphatic system may be harnessed to treat T2D and cardiovascular diseases associated with obesity and insulin resistance.

## The Lymphatic System

### Lymphatic Structure

**FIGURE 1:**
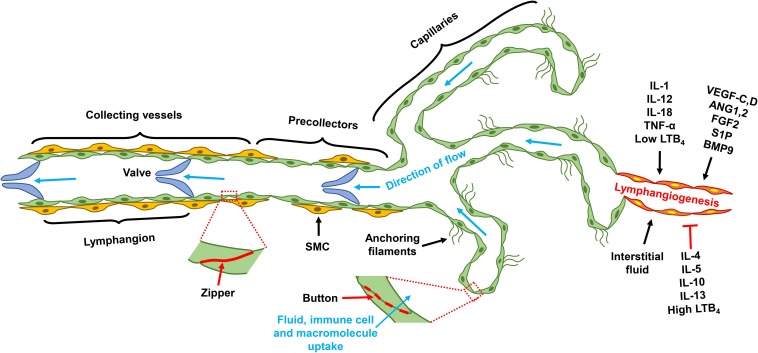
Lymphatic vascular tree and lymphangiogenesis. Lymphatic capillaries are comprised of single layer LECs that tether directly to the extra cellular matrix. Junctional proteins form button structure that interconnects capillary LECs; and those buttons allow the formation of overlapping LEC flap, through which interstitial fluid and macromolecule enter the blind-ends of the capillaries. Capillaries converge into pre-collectors, which in turn coalesces into collecting lymphatics. SMCs loosely cover the precollector, but invest collecting lymphatics more completely. Junctional proteins of the collecting lymphatics form a continuous structure, known as zipper. Collecting lymphatics are comprised of lymphangions that are demarcated by intravascular valves, the segment between two valves is designated as one lymphangion. Factor regulates lymphangiogenesis include pro-lymphangiogenic growth factors, pro-inflammatory cytokines, and interstitial fluid. Type 2 cytokines, and high concentrations of LTB_4_, are molecules that suppress lymphangiogenesis. LEC, lymphatic endothelial cell; VEGF, vascular endothelial growth factor; ANG, angiopoietin; FGF, fibroblast growth factor; S1P, sphingosine 1 phosphate; BMP, bone morphogenetic protein; IL, interleukin; LTB_4_, leukotriene B_4_; TNF, tumor necrosis factor; SMC, smooth muscle cell.

The lymphatic system is comprised of lymphatic vessels and the secondary lymphoid organs that include lymph nodes, spleen, tonsils, and Peyer’s patches (Ruddle and Akirav, 2009; Choi et al., 2012). The lymphatic vasculature is a unidirectional circulatory network that begins as blunt-ended capillaries composed of a single layer of lymphatic endothelial cells (LECs) that tether directly to the interstitial tissue through anchoring filaments, with discontinuous basement membrane coverage (Jiang et al., 2018). Capillary LECs are interconnected by unique button-like structures that are formed by discontinuous layers of junctional proteins such as VE-cadherin, claudin, Zonula occludens-1 (ZO-1), connexin, and occludin (Baluk et al., 2007). The presence of these buttons serves to create overlapping LEC flaps that serve as *de facto* primary valves. In response to interstitial fluid pressure fluctuation, these flaps can open and close to regulate fluid reabsorption as well as the uptake of macromolecules and immune cells (Yao et al., 2012; Jiang et al., 2018). The lymphatic capillaries converge into precollectors and these in turn coalesce into the collecting lymphatics, in which LECs are joined by zipper-like, continuous, seamless junctions and invested with basement membrane along with smooth muscle cell coverage. Intraluminal lymphatic valves divide the collecting lymphatics into contractile segments designated as lymphangions, thus providing a structural basis for the unidirectional lymph flow (Aspelund et al., 2016; Figure 1). The collecting lymphatics travel through chains of lymph nodes, which allows the delivery of free antigens and antigen-loaded dendritic cells (DCs) from interstitial tissue for immune priming; the central lymphatic vasculature eventually joins the subclavian veins via the thoracic duct(s) conveying the lymph node-filtered interstitial fluid back to the blood circulatory system (Thomas et al., 2016; Jiang et al., 2018).

### Early Lymphatic Development and Lymphangiogenesis

Lymphatic vasculature originates from the cardinal vein and subsequently develops independently from the blood circulatory system (Wigle and Oliver, 1999). The homeobox gene *Prox-1* appears to be the master control gene for lymphatic differentiation and development (Choi et al., 2012). In E9.5 mouse embryos, Prox-1 expression begins in a subset of endothelial cells (ECs) of the cardinal vein and specifies them as LECs. Prox-1 then upregulates lymphatic markers such as LYVE-1, VEGFR3, and Chemokine (C–C motif) ligand (CCL)21, and concurrently downregulates blood vascular signature genes (Wigle et al., 2002). This molecular differentiation enables LEC budding from the cardinal vein to form the rudimentary lymphatic vessels, known as the jugular lymph sac, at E11.5 (Choi et al., 2012). Conditional Prox-1 downregulation reprograms LECs into blood endothelial cells (BECs) in both developing and adult mice as well as in cell culture (Johnson et al., 2008), supporting the notion that Prox-1 is an indispensable transcriptional factor for the maintenance of LEC identity. Following the initial specification and budding, the lymph sac then expands through lymphangiogenesis, a process of new lymphatic vessel sprouting from preexisting structures (Aspelund et al., 2016). VEGF-C/D-activated VEGFR3 signaling is the most central pathway for lymphangiogenesis (Zheng et al., 2014). VEGF-C deficiency leads to lymphatic insufficiency and lymphedema, a defect that can be rescued by VEGF-D during development (Karkkainen et al., 2004); VEGFR3 activation is also critical for pathophysiological lymphangiogenesis following lymphatic injury (Szuba et al., 2002). VEGFR3 missense mutations in the tyrosine kinase domains underlie the etiology of 70% cases of primary congenital lymphedema, known as Milroy disease (Karkkainen et al., 2000; Connell et al., 2009), indicating that the VEGFR3 signaling is important for the normal development of human lymphatic vasculature. The axon guidance protein neuropilin (NRP)-2 enhances VEGFR3 signaling by acting as its co-receptor (Yuan et al., 2002; Xu et al., 2010). By facilitating VEGF-C maturation, the collagen- and calcium-binding EGF domains 1 (CCBE1) protein also promotes lymphangiogenesis (Bos et al., 2011; Jha et al., 2017). Thus, both NRP2 and CCBE1 are factors involved in lymphangiogenesis by regulating the VEGF-C/VEGFR3 signaling.

Following the initial sprouting, lymphatic capillaries mature through activating the Notch signaling pathway (Zheng et al., 2011), resembling the well-known tip–stalk cell paradigm in angiogenesis (Potente et al., 2011). Maturation of collecting lymphatic vessels requires concerted smooth muscle cell recruitment and patterning as well as valve development. Although angiopoietin (ANG)2 antagonizes TIE2 receptor activation during blood angiogenesis, it appears to stimulate TIE2 signaling in LECs and to promote postnatal lymphatic remodeling (Gale et al., 2007); ANG2-deficient mice do not develop normal hierarchical lymphatic vascular system (Dellinger et al., 2008). The forkhead box protein FOXC2 modulates lymphatic capillary development by controlling SMC recruitment and basement membrane formation (Petrova et al., 2004). In coordination with Prox-1, FOXC2 senses lymph flow and induces the expression of gap junction protein connexin 37 (Cx37) and activates calcineurin/nuclear factor of activated T-cells (NFAT) signaling, which regulates lymphatic valve formation (Sabine et al., 2012). While FOXC2 controls ANG2 expression in angiogenesis (Xue et al., 2008), it induces TIE2 in LECs (Thomson et al., 2014), suggesting that FOXC2 may promote lymphatic maturation by regulating the ANG2/TIE2 signaling.

Other signaling pathways, including fibroblast growth factor (FGF)2/FGF receptor (FGFR)1, sphingosine-1-phosphate (S1P)/S1P receptor (S1PR)1, and bone morphogenetic protein (BMP)9/activin receptor-like kinase 1 (ALK1), also regulate lymphangiogenesis (Zheng et al., 2014). Additionally, pro-inflammatory cytokines influence lymphangiogenesis and affect immune activation and resolution. In general, type 1 cytokines including IL-1, IL12, IL-18, and TNF-α promote, and type 2 and anti-inflammatory cytokines such as IL-4, IL-5, IL13, and IL-10 suppress lymphangiogenesis (Sainz-Jaspeado and Claesson-Welsh, 2018). The pro-inflammatory lipid molecule leukotriene B4 (LTB_4_) promotes lymphatic regeneration at low concentration but suppresses lymphangiogenesis at high pathological concentrations (Tian et al., 2017). In summary, lymphangiogenesis is regulated by growth factors and cytokines, as well as by interstitial fluid flow (Figure 1).

## The Interplay Between Lymphatic Vasculature and Obesity

Interaction of genetic, epigenetic, environmental, and psychological factors regulates the production of physiological mediators that control the balance of energy intake and expenditure (Gonzalez-Muniesa et al., 2017). When energy intake is in surplus, about 70–80% of the excessive intake is stored as fat, and the remainder is converted into glycogen or protein or lost as heat; long-term positive energy balance ultimately leads to obesity (Oussaada et al., 2019). Genetic mediation of obesity can be monogenic or polygenic. Monogenic mutation is relatively rare and primarily affects the genes involved in the leptin–melanocortin pathway, the central regulator of food intake and energy balance (Oussaada et al., 2019); as examples, the leptin receptor (LEPR) (Farooqi et al., 2007), pro-opiomelanocortin (POMC) (Krude et al., 2003), melanocortin 4 receptor (MC4R) (Krude et al., 2003), and MC3R (Lee et al., 2002) have all been linked to early onset of obesity in humans. Polygenic causation, however, is more common and accounts for >90% cases of childhood-onset obesity (Kleinendorst et al., 2018). Notably, common variants in certain loci within the fat mass and obesity-associated gene (FTO) have been linked to higher BMI in human populations (Claussnitzer et al., 2015; Oussaada et al., 2019). DNA methylation studies have identified epigenetic modification of several genes that are associated with obesity, as detailed elsewhere (Rohde et al., 2019). Whether obesity promotes epigenetic change and further deteriorates the imbalance between energy intake and expenditure to exacerbate obesity is an open question.

The lymphatic vasculature regulates both dietary lipid absorption and peripheral cholesterol removal. The intestinal lacteals are lymphatic vessels comprised of both capillary and collecting lymphatic elements. The lacteals absorb dietary lipids packaged as chylomicrons (Iqbal and Hussain, 2009; Randolph and Miller, 2014). Intravital imaging indicates that lacteals possess a spontaneous contractile feature; they actively absorb and transport enterocyte-processed lipids to the systemic circulation in concert with contractile forces produced by adjacent smooth muscle cells controlled by the autonomic nervous system (Choe et al., 2015). It was recently revealed that lacteal function controls dietary lipid absorption and, consequently, body weight, supporting the concept that the lacteals are the gatekeepers of lipid intake from the environment (McDonald, 2018; Zhang et al., 2018; Cifarelli and Eichmann, 2019).

In peripheral tissues, the lymphatic vasculature is generally considered to be the only route for the return of lipoprotein to the blood circulation (Cooke et al., 2004); removal of interstitial cholesterol by the lymphatic route is known as reverse cholesterol transport (RCT) (Huang et al., 2015). Although both lacteals and peripheral lymphatics may selectively uptake cargoes based on their size (Randolph and Miller, 2014), they may preferentially absorb lipoproteins depending on specific receptor expression. As an example, LEC expression of the scavenger receptor class B type I (SR-B1) is required for RCT in skin lymphatic capillaries but not for intestinal cholesterol absorption (Bura et al., 2013; Lim et al., 2013). Impaired RCT has been observed in ob/ob mice with induced obesity (Duong et al., 2018), suggesting that defective RCT may be a prerequisite for the development of obesity.

Several lines of evidence support the notion that lymphatic functionality impacts the pathogenesis of obesity. In patients with lymphatic injury-induced (secondary) lymphedema, fat hypertrophy in the lymphedematous tissues is prominent, accompanying tissue swelling and fibrosis (Jiang et al., 2018); the Chy mutant mouse, with its defective lymphatic development, also display abnormal lipid accumulation adjacent to affected hypoplastic lymphatic vessels (Karkkainen et al., 2001). *Prox-1* haploinsufficiency causes lymphatic dysfunction and leads to adult-onset of obesity (Harvey et al., 2005), in those mice, lymphatic restoration rescues them from the development of obesity (Escobedo et al., 2016). Accumulated interstitial fluid, including retrograde lymph leakage from the dysfunctional lymphatics, may promote adipocyte differentiation and enhance local fat deposition (Harvey et al., 2005; Escobedo and Oliver, 2017). Increasing lymphatic density in adipose tissue by overexpressing VEGF-D reduces local immune cell accumulation and improves systemic metabolic responsiveness in high-fat diet (HFD)-induced obese mice (Chakraborty et al., 2019), although it cannot be excluded that VEGF-D may also exert effects on blood vascular cells because it also binds to VEGFR2 (Achen et al., 1998). Increased expression of Apelin, an endogenous peptide identified as a ligand of the orphan G protein-coupled receptor APJ, was shown to inhibit HFD-induced obesity by promoting both lymphatic and blood vascular integrity (Sawane et al., 2013). Collectively, these studies suggest that lymphatic dysfunction promotes obesity, and that improving lymphatic function inhibits the development of obesity and alleviates obesity-caused metabolic syndrome. Studies have also illustrated that obesity promotes lymphatic abnormalities, such as decreased initial lymphatic density, heightened lymphatic leakiness, impaired collecting lymphatic pumping, and diminished macromolecule transport; but those phenotypic and functional changes are reversible in response to dietary modification and weight control (Garcia Nores et al., 2016; Nitti et al., 2016). Decreased collecting lymphatic pumping may result from the perilymphatic accumulation of iNOS-expressing macrophages that can affect lymphatic contractility and damage LECs by conversion of NO to a powerful oxidant, peroxynitrite (Nitti et al., 2016; Torrisi et al., 2016). Consistent with experimental animal data, a clinical study showed that obesity is a risk factor for the development of lymphedema in post-surgical breast cancer patients (Helyer et al., 2010). Also, severely obese individuals often develop acquired lymphedema of the extremities (Greene et al., 2012). *In vitro* cell culture study showed that leptin compromises LEC proliferation and tube formation by enhancing STAT3 phosphorylation, although leptin also induces IL-6, which, on the other hand, promotes lymphatic tube formation. In aggregate, the net effect of high concentrations of leptin on lymphangiogenesis appears to be suppressive (Sato et al., 2016). These findings suggest that high concentrations of leptin produced by adipose tissue maybe responsible for suppressing lymphatic vasculature in obese individuals. In summary, lymphatic dysfunction sensitizes individuals to develop obesity, and obesity worsens lymphatic function.

## Lymphatic Endothelial Cell Insulin Resistance

Insulin signaling regulates glucose, lipid, and energy homeostasis, predominantly through its action on adipose tissues, liver, and skeletal muscles (Boucher et al., 2014). Insulin exerts its known function by binding to the insulin receptor (INSR) expressed on target cells; ligand engagement leads to INSR autophosphorylation, followed by recruitment of various phosphotyrosine-binding scaffold proteins, which in turn activates downstream effectors (Taniguchi et al., 2006). The most crucial INSR substrates for metabolic regulation are insulin receptor substrate (IRS)1 and IRS2 proteins. They exert downstream effects by activating the PI3K/AKT signaling pathway (Petersen and Shulman, 2018). Insulin signaling in skeletal muscle promotes glucose uptake and net glycogen synthesis; IRS1 appears to be the primary scaffold protein for this process (Bouzakri et al., 2006; Thirone et al., 2006). In the liver, insulin promotes the synthesis of major classes of metabolic macromolecules, including glycogen, lipids, and proteins, and, concurrently, it reduces hepatic glucose production by controlling the PI3K/AKT/GSK3α/β or mTORC1 or FOXO1 signaling cascades (Cherrington et al., 1998; Petersen and Shulman, 2018). In white adipose tissue (WAT), insulin signaling suppresses lipolysis; but its role in glucose uptake is relatively minor, accounting for about 5–10% of whole body glucose uptake (Virtanen et al., 2002; Ng et al., 2012). Attenuation or reversal of cAMP/PKA-mediated lipolysis induced by adrenergic signaling is the best understood mechanism for insulin suppression of lipolysis (Jaworski et al., 2007). Insulin resistance arises under the condition of chronic energy surplus (Samuel and Shulman, 2016). Continuous overnutrition with insulin resistance impairs insulin secretion by pancreatic β-cells, which eventually leads to overt T2D (DeFronzo et al., 2015). At the molecular level, both decreased INSR expression and impaired intracellular signaling transduction contribute to typical obesity-induced insulin resistance (Petersen and Shulman, 2018).

**FIGURE 2:**
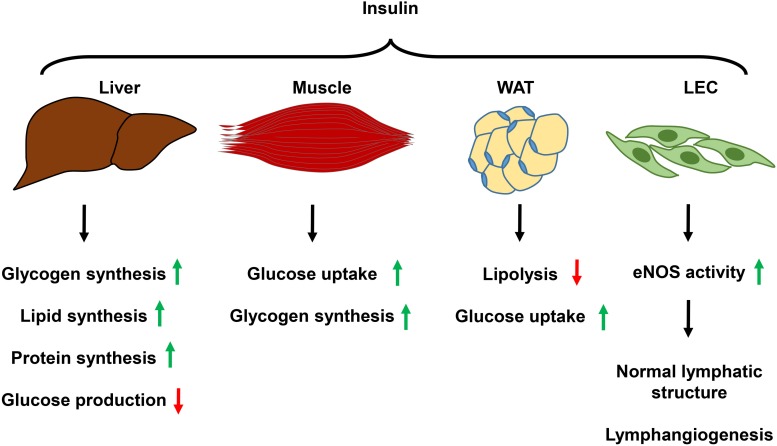
Summary of the function of insulin signaling in metabolic organs and LECs. Insulin signaling in the liver promotes glycogen, lipid, and protein synthesis while decreases glucose production. In the skeletal muscle, insulin mediates glucose uptake and glycogen synthesis. In white adipose tissue, insulin suppresses lipolysis and promotes glucose uptake. Insulin activation of its receptor in LECs promotes eNOS activity, which keeps normal lymphatic structure, and promotes lymphangiogenesis. WAT, white adipose tissue; LEC, lymphatic endothelial cell; eNOS, endothelial nitric oxide synthase.

While WAT, liver, and skeletal muscles have been generally recognized as major insulin target tissues, ECs, including both BECs and LECs, are also sensitive to insulin. It was suggested that blood vascular ECs might act as “first-responders” to overnutrition (Barrett and Liu, 2013). Impaired insulin signaling in BECs diminishes AKT-dependent NO production and simultaneously increases Endothelin 1 (ET-1) activity, which leads to endothelial dysfunction (Mather et al., 2002; Federici et al., 2004; Okon et al., 2005; Shemyakin et al., 2006; Muniyappa and Sowers, 2013). Unhealthy microvasculature hampers insulin delivery to muscle and adipose tissue and affects glucose disposal and lipid homeostasis (Barrett and Liu, 2013). LEC insulin signaling has only recently been explored. One study showed that LECs derived from human dermal tissue (HDLECs) express much higher levels of INSR than that of adipose tissue microvascular ECs; an insulin level as low as 2.5 nM can induce AKT phosphorylation in HDLEC (Jaldin-Fincati et al., 2018), although circulating insulin concentrations in healthy individuals may still well-below the 2.5 nM range, which are around 100 pmol/L (Ford et al., 2006), whether similar responses can be induced at physiological conditions are unknown. Insulin-induced downstream signaling appears to be required for normal lymphatic vascular structure and function (Lee et al., 2018). Diminished LEC insulin signaling decreases eNOS phosphorylation and NO production, reduces mitochondria oxygen consumption, which alters LEC metabolism, and causes increased expression of proinflammatory molecules (Lee et al., 2018); these results suggest that physiological insulin signaling is essential for normal functioning of LECs. Supporting the role of insulin signaling in lymphatic function, blockade of IRS1 suppresses lymphangiogenesis (Hos et al., 2011). Collectively, these studies indicate that insulin signaling likely plays important roles in regulating both LEC metabolism and lymphangiogenesis (Figure 2); LEC insulin resistance diminishes lymphatic function, and exacerbates obesity and metabolic abnormality.

## Lymphatic Vascular Pathophysiology in T2D

Type 2 diabetes is characterized by dysregulation of carbohydrate, lipid, and protein metabolism caused by impaired insulin signaling resulting from reduced insulin secretion, insulin resistance, or the combination of both (DeFronzo et al., 2015). The lymphatic pathology likely co-evolves with the pathogenesis of diabetes. In an alloxan-induced rat diabetes model, the lymph flow through the thoracic duct in diabetic rats is significantly higher than in that of healthy controls, possibly because increased interstitial glucose levels increase tissue colloid pressure, which then enhances interstitial fluid absorption and lymph production. By contrast, lymph node uptake of ^99*m*^Tc−dextran 500 is impaired in diabetic rats, a result that might explain the observation that patients with diabetes often have decreased function for immune priming (Moriguchi et al., 2005). Interestingly, insulin treatment normalizes both lymph flow and lymph node dextran retention, but glucose normalization through diet-control only corrects lymph flow (Moriguchi et al., 2005), suggesting that fluid transport and immune regulatory function of the lymphatics are regulated by discrete mechanisms. It is worth noting that the alloxan model simulates type 1 diabetes (Ighodaro et al., 2017), it nevertheless will provide insights about how hyperglycemia may impact the lymphatic system. In a clinical study, skin specimens of diabetic patients displayed increased lymphatic density, and transcriptional analysis of isolated dermal LECs indicated that cells from diabetic patients exhibit signatures of inflammation, adhesion, migration, growth, and lymphangiogenesis (Haemmerle et al., 2013). Increased lymphatic density found in human patient samples seemingly correlates with the phenomenon observed in diabetic rats that have enhanced lymphatic return to the systemic circulation. However, it is possible that the lymphatic function may decompensate after prolonged interstitial fluid overload caused by hyperglycemia (Kanapathy et al., 2015). Increased lymphatic collecting vessel permeability caused by diminished NO availability appears to be a contributing factor for lymphatic dysfunction (Scallan et al., 2015).

One common complication of diabetes is diabetic retinopathy, which is characterized by a pathology that involves vascular, glial, and neuronal components and causes significant visual loss (Duh et al., 2017). A recent study investigating the lymphatics in diabetic retinopathy detected the expression of LEC markers, such as VEGFR3 and Prox-1, in excised human specimens (Loukovaara et al., 2015), providing the first clinical evidence that abnormal lymphatic growth, in addition to pathological microvascular remodeling, occurs in diabetic retinopathy. A follow-up study demonstrated that soluble pro-growth factors in vitreous fluid promoted lymphatic sprouting of patient-derived tissues with diabetic retinopathy (Gucciardo et al., 2018a), further supporting the notion that the microenvironment of the eyes of diabetic retinopathy contains sufficient cue for abnormal lymphatic expansion. Pathological lymphangiogenesis observed in the eyes of diabetic retinopathy may, therefore, be regarded as a feature for therapeutic targeting (Yang et al., 2016; Gucciardo et al., 2018b). Ongoing clinical trials targeting VEGFR- or RTK-mediated signaling may provide possibilities to suppress pathological lymphatic overgrowth, but identification of novel lymphatic specific targets is probably necessary for better therapeutic outcomes (Williams et al., 2010).

## Lymphatics in Atherosclerosis and Myocardial Infarction

The metabolic syndrome poses a significant risk for the development of cardiovascular diseases, such as atherosclerosis and its severe complication, myocardial infarction (MI) (Wilson et al., 2005; Mottillo et al., 2010). Following the revelation of the interplay between metabolic syndrome and lymphatic dysfunction, a multiplicity of research has shown that the lymphatic vasculature is also actively involved in the progression of atherosclerosis and development of MI (Aspelund et al., 2016). Atherosclerosis is a chronic inflammatory disease of the arterial wall with dyslipidemia as the root cause. The atherosclerotic lesion is characterized by a prominent population of lipid-filled foam cells, which are macrophages that contain an excess of plasma-derived, modified lipoproteins (Back et al., 2019). In this inflammatory pathology, the balance between pro- and anti-inflammatory molecules dictates whether a nascent atherosclerotic lesion will reverse course to normal or progress to more advanced stages. Among all the inflammatory cells that populate an atherosclerotic locus, the lipid-laden macrophage is central to disease progression. Macrophage uptake and modification of lipoprotein are physiologically important for lipid removal from intima, but uncontrolled, excessive lipid infiltration and inflammation exhausts macrophages and creates a hypoxic environment; this, in turn, promotes macrophage death and release of oxidized lipid and perpetuates the vicious cycle of foam cell formation, death, and intimal lipid accumulation (Tabas, 2010). Tipping the balance of local inflammation to an anti-inflammatory profile is, therefore, a therapeutic concept in treating atherosclerosis (Ruparelia et al., 2017). Recent studies have delved into lymphatic biology in the pathogenesis of atherosclerosis, presumably because the lymphatics play an essential role in immune trafficking and lipid transport (Milasan et al., 2015; Csanyi and Singla, 2019).

Lymphatic vessels are present in the adventitial and periadventitial regions of arterial walls (Sacchi et al., 1990; Martel et al., 2013; Milasan et al., 2015). Early observations that associated diminished lymphatic drainage to atherosclerosis were reported nearly three decades ago (Miller et al., 1992; Solti et al., 1994). Utilizing a mouse aorta transplantation model, the lymphatic vasculature was shown to be critical for RCT in arterial wall of the large vessel (Martel et al., 2013). Another study demonstrated that mice fed a HFD were prone to atheroma formation when the lymphatic vasculature is defective (Vuorio et al., 2014), supporting the notion that lymphatic dysfunction diminishes cholesterol removal and promotes atherosclerosis. In atherosclerosis-prone *Ldlr*^–/–^; *ApoB100*^+/+^ mice, lymphatic dysfunction, mainly of the collecting lymphatic vessels, occurs before the onset, and during the progression, of atherosclerosis (Milasan et al., 2016), suggesting that lymphatic vascular abnormalities likely promote atherosclerosis. In agreement with these hypotheses, the rescue of lymphatic vasculature during the early phase of atherogenesis retards disease progression, through reducing tissue inflammation and, likely also through increased lymphatic cholesterol transportation (Milasan et al., 2019). These studies in aggregate link lymphatic vascular functionality to atherosclerosis pathogenesis.

While the lymphatic vasculature is critical for immune trafficking and immune regulation, inflammatory molecules, produced by infiltrated local immune cells, also impact lymphatic vascular remodeling and function. Targeting inflammation may, therefore, not only ameliorate tissue inflammation but also improve lymphatic function. In a mouse tail surgery-induced tail lymphedema model, we have previously shown that high concentrations of LTB_4_ sustain local tissue inflammation, which is characterized by infiltration of cells of both innate and adaptive immunity; blockade of LTB_4_ signaling not only reduces tissue inflammation but also improves lymphatic function and alleviates lymphedema (Tian et al., 2017). In a series of *in vivo* and *in vitro* experiments, we showed that >100 nM LTB_4_ induces LEC apoptosis and suppresses lymphangiogenesis, while LTB_4_ in the 10 nM range enhances lymphatic regeneration; a physiological LTB_4_ level appears to be essential for surgery-induced wound healing (Tian et al., 2017); these experiments indicate that inflammation and lymphatic dysfunction are closely associated. LTB_4_ is an important proinflammatory molecule that promotes atherosclerosis by magnifying monocyte chemotaxis and foam cell formation (Subbarao et al., 2004), LTB_4_ may also directly impact endothelial survival and angiogenesis (Tian et al., 2013) and exacerbates insulin resistance (Li et al., 2015). It is therefore possible that targeting the LTB_4_ signaling pathway may simultaneously limit tissue inflammation, boost both lymphatic and blood vasculature, increase insulin sensitivity, and normalize cellular metabolism. Several Phase I atherosclerosis clinical trials targeting the LTB_4_ pathway are underway (Bhatt et al., 2017).

Myocardial infarction can be triggered by rupture or erosion of vulnerable atherosclerotic plaque, which results in blood clot formation by the exposure of thrombogenic core and matrix components of plaque (Anderson and Morrow, 2017). MI is followed by robust inflammatory responses that can be categorized into the initial pro-inflammatory responses that function to remove necrotic debris, and reparative anti-inflammatory responses poised to repair the damaged tissue (Ong et al., 2018). Targeting the inflammatory response in different phases of MI appears to be logical, but several such interventions have failed to improve patient outcomes (Ong et al., 2018), indicating additional mechanisms are likely at play for post-MI recovery. A recent study revealed significant lymphangiogenic responses following MI. Promotion of lymphangiogenesis by exogenous administration of VEGFC results in a transient improvement of post-MI myocardial function (Klotz et al., 2015). Improved outcome by VEGFC-induced lymphangiogenesis depends on lymphatic-mediated immune cell clearance through a pathway involving LEC expressed LYVE-1 (Vieira et al., 2018). Consistent with a protective role of the lymphatic vasculature in promoting post-MI recovery, downregulation of the LEC marker VEGFR3 alters cardiac lymphatic structure, increases lymphatic leakage, and raises MI-induced mortality (Vuorio et al., 2018). These recent studies suggest that the lymphatic vasculature might be a viable therapeutic target for post-MI cardiac repair (Vuorio et al., 2017).

## Concluding Remarks

Type 2 diabetes and cardiovascular diseases associated with obesity are the leading cause of death in the developed world (Benjamin et al., 2019). There is an unmet need to improve the medical care for these patients. Our understanding of mechanisms underlying these diseases has grown substantially but remains incomplete. Promoting lymphatic function has the apparent capacity to reduce pathology in preclinical obesity and cardiovascular disease models. More in-depth study of lymphatic biology is therefore urgently needed. Discoveries that derive from these investigations will likely provide novel therapeutic targets and improve disease survival.

## Author Contributions

XJ contributed to the writing and editing of the manuscript, and creation of figures. WT and MN contributed to the concepts and editing of the manuscript. SR contributed to the concepts, editing, and final formatting of the manuscript.

## Conflict of Interest

The authors declare that the research was conducted in the absence of any commercial or financial relationships that could be construed as a potential conflict of interest.
